# Modeling the Effect of HIV/AIDS Stigma on HIV Infection Dynamics in Kenya

**DOI:** 10.1007/s11538-021-00891-7

**Published:** 2021-04-05

**Authors:** Ben Levy, Hannah E. Correia, Faraimunashe Chirove, Marilyn Ronoh, Ash Abebe, Moatlhodi Kgosimore, Obias Chimbola, M. Hellen Machingauta, Suzanne Lenhart, K. A. Jane White

**Affiliations:** 1 Department of Mathematics, Fitchburg State University, Fitchburg, MA USA; 2 Harvard Data Science Initiative, Harvard University, Cambridge, MA USA; 3 Department of Biostatistics, Harvard University, Boston, MA USA; 4 School of Mathematics, University of Nairobi, Nairobi, Kenya; 5 Department of Mathematics and Applied Mathematics, University of Johannesburg, Johannesburg, South Africa; 6 Department of Biometry and Mathematics, Botswana University of Agriculture and Natural Resources, Gaborone, Botswana; 7 Mathematics and Statistical Sciences, Botswana International University of Science and Technology, Palapye, Botswana; 8 Mathematics Department, University of Tennessee, Knoxville, TN USA; 9 Department of Mathematical Sciences, University of Bath, Bath, UK

**Keywords:** HIV, Stigma, Kenya, Mathematical model, UN goals

## Abstract

Stigma toward people living with HIV/AIDS (PLWHA) has impeded the response to the disease across the world. Widespread stigma leads to poor adherence of preventative measures while also causing PLWHA to avoid testing and care, delaying important treatment. Stigma is clearly a hugely complex construct. However, it can be broken down into components which include internalized stigma (how people with the trait feel about themselves) and enacted stigma (how a community reacts to an individual with the trait). Levels of HIV/AIDS-related stigma are particularly high in sub-Saharan Africa, which contributed to a surge in cases in Kenya during the late twentieth century. Since the early twenty-first century, the United Nations and governments around the world have worked to eliminate stigma from society and resulting public health education campaigns have improved the perception of PLWHA over time, but HIV/AIDS remains a significant problem, particularly in Kenya. We take a data-driven approach to create a time-dependent stigma function that captures both the level of internalized and enacted stigma in the population. We embed this within a compartmental model for HIV dynamics. Since 2000, the population in Kenya has been growing almost exponentially and so we rescale our model system to create a coupled system for HIV prevalence and fraction of individuals that are infected that seek treatment. This allows us to estimate model parameters from published data. We use the model to explore a range of scenarios in which either internalized or enacted stigma levels vary from those predicted by the data. This analysis allows us to understand the potential impact of different public health interventions on key HIV metrics such as prevalence and disease-related death and to see how close Kenya will get to achieving UN goals for these HIV and stigma metrics by 2030.

## Introduction

HIV/AIDS-related stigma and discrimination continue to impede the progress of *responses to* HIV/AIDS across the world (Chesney and Smith 1999). While the percentage of people expressing discriminatory attitudes toward people living with HIV/AIDS has decreased over time, on average more than half of adults in 36 countries across the globe still express discriminatory attitudes (ICF 2018). People living with HIV/AIDS (PLWHA) who experience high levels of HIV/AIDS-related stigma avoid testing and delay initiating HIV/AIDS care and treatment (Golub and Gamarel 2013; Price et al. 2019; Remien et al. 2015; Ti et al. 2013; Treves-Kagan et al. 2017). Further, individuals living with HIV/AIDS *avoid* frequenting hospitals for treatment or collecting antiretroviral therapy (ART) drugs for fear of health workers disclosing their HIV/AIDS status to the communities (Kagee et al. 2011; Mills et al. 2006). Available data across 19 countries confirm that one in four PLWHA face discrimination in health care (Global Network of People with HIV/AIDS and International Community of Women living with HIV/AIDS 2017), and one in five avoid healthcare treatment due to fear of discrimination (King et al. 2013; Nyblade et al. 2017). Approximately one in every eight PLWHA are denied health care due to stigma regarding their status, and women living with HIV/AIDS face greater discrimination in health care than their male counterparts (Global Network of People with HIV/AIDS and International Community of Women living with HIV/AIDS 2017). Stigma or fear of stigma results in poor adherence to pre-exposure prophylaxis and antiretroviral therapy, leading to high HIV/AIDS viral loads (Buregyeya et al. 2017; Croome et al. 2017; Katz et al. 2013; Patel et al. 2016). Stigmatized PLWHA are also less likely to disclose their HIV/AIDS status to their sex partner(s) (McKay and Mutchler 2011).

In sub-Saharan Africa, rates of HIV/AIDS-related stigma remain particularly high, and so do infection levels. In Kenya, a peak in new HIV infections in 1995 was followed by a peak in deaths attributed to HIV/AIDS in 2004, and although numbers of new HIV infections are falling, the decrease has been no more than 1000 individuals per year since 2010 (Global Burden of Disease Collaborative Network 2018).

At the United Nations (UN) General Assembly Special Session on HIV/AIDS in 2001, African governments agreed to combat all forms of discrimination against PLWHA and subsequently the UN released *the* “*Getting to Zero*” initiative in 2011. The goals of this initiative were to get new infections, discrimination, and deaths from HIV/AIDS to zero by 2030, clearly recognizing the importance of reductions in both infection and stigma levels in order to achieve the ambitious goal. However, HIV/AIDS-related stigma and discrimination are difficult to overcome solely through top-down initiatives and messaging campaigns (Campbell and Cornish 2010; Johnny and Mitchell 2006; Parkhurst 2014) and while there has been progress, it seems unlikely that the zero goals will be achieved.

Many researchers have formulated mathematical models to understand the dynamics of HIV/AIDS. We are aware of work on epidemiological models for HIV infection levels and spread in Africa (Nyabadza et al. 2011; Simwa and Pokhariyal 2003), including some models that consider interventions such as treatment, use of condoms, and contact tracing (Hyman et al. 2003; Moghadas et al. 2003; Granich et al. 2009). Some models include features representing information that causes changes in the behavior of individuals living in a society with strong HIV prevalence (Joshi et al. 2008; Ronoh et al. 2020). However, there are very few examples that include stigma explicitly within infectious disease dynamic models. We call attention to a system of four ODEs used for showing dynamics and game theoretical results illustrating interactions of stigmatization and prevalence in a generic infectious disease (Reluga et al. 2019). Two recent papers used structural equation modeling and cohort scenario analysis to examine the effects of stigma on African women with HIV (Logie et al. 2016; Prudden et al. 2017).

Here, we seek to investigate the effects of HIV/AIDS-related stigma on the dynamics of an HIV infection model which includes a class of infected individuals that are receiving treatment. We specifically focus on understanding the effects of stigma on HIV/AIDS dynamics in Kenya which has some of the highest estimated prevalence of HIV/AIDS in the world (UNAIDS 2018). Our model approach has two strands. First we use survey data to create a time-varying measure of stigma in the adult population of Kenya; we use that to build a model for stigma which feeds into our compartmental model for HIV infection dynamics.

We begin in Sect. 2, by establishing a model for population stigma, parameterizing it using data from Kenya Demographic and Health surveys (CBS et al. 2004; Kenya National Bureau of Statistics (KNBS) and ICF Macro 2010, 2014). This feeds into a compartmental model for HIV infection in Kenya and the associated parameter estimation in Sect. 3. Our results, presented in Sect. 4, explore how baseline stigma parameters impact infection prevalence and HIV-related deaths. We modify the parameter estimates to undertake a numerical exploration focussed on understanding how changes to internalized versus enacted stigma would have impacted HIV infection measures; we complement this with a simple steady-state analysis to gain insight into how the infection dynamics evolve. In the final section, we discuss our results in the context of the impact of stigma on HIV dynamics in Kenya highlighting the urgent need to gather more data on stigma and its associated impact on HIV dynamics.

## Modeling Population Stigma

Stigma is a socially devalued attribute that gives rise to social inequality in the form of labeling, stereotyping, devaluation, status loss, or discrimination arising from the social judgment applied to a person or group who possesses the devalued attribute (Earnshaw and Chaudoir 2009; Van Brakel 2006). It keeps those with a socially devalued attribute in a position of relative subordination to those without the devalued attribute (Link and Phelan 2001; Parker and Aggleton 2003).

One of the main approaches to measuring HIV/AIDS-related stigma is the assessment of discriminatory attitudes, including measures calculated from questions regarding a person’s potential actions toward a PLWHA (Van Brakel 2006; Earnshaw and Chaudoir 2009). Select studies have also measured stigma through interviews with PLWHA asking how many times or how often they have experienced various forms of discrimination over the past year (Neuman and Obermeyer 2013). Indices for HIV/AIDS-related stigma have been developed previously, however most were intended for use in the USA and few have been broadly deployed (Van Brakel 2006).

PLWHA experience stigma through three mechanisms (Earnshaw and Chaudoir 2009; Van Brakel 2006):

- enacted or experienced stigma;
- anticipated or perceived stigma; and
- internalized stigma.

There are two stages to our modeling activity. Firstly, we estimate population-level stigma in Kenya using data from the Kenya Demographic and Health Surveys (KDHS) from 2003, 2009, and 2014. This results in only three data points which are insufficient to make accurate predictions. However, the points allow us to predict parameters of our dynamic model for stigma. In the second stage, we create a simple linear model to describe the change in stigma over time using mechanistic principles and guided by Occam’s Razor.

### Obtaining Data Points for Stigma in Kenya

The KDHS from 2003, 2009, and 2014 provide data on HIV/AIDS knowledge, relevant behavior, and attitudes toward PLWHA captured at the national level and for demographically homogeneous subpopulations (CBS et al. 2004; Kenya National Bureau of Statistics (KNBS) and ICF Macro 2010, 2014). The questionnaire module on attitudes toward PLWHA asks survey respondents familiar with AIDS the following four questions:

1. Would you buy fresh vegetables from a shopkeeper or vendor if you knew that this person had HIV?
2. If a member of your family became sick with AIDS, would you be willing to care for her or him in your own household?
3. In your opinion, if a female teacher has the AIDS virus, but is not sick, should she be allowed to continue teaching in the school?
4. If a member of your family got infected with the AIDS virus, would you want it to remain a secret or not?

The first three questions capture the stigma mechanism of social distancing from PLWHA, while the fourth question aims to measure perceived or anticipated stigma enacted by others should the respondent be associated with HIV/AIDS (Chan and Tsai 2016). Question 2 in the DHS has been found to be interpreted very differently by men and women and so is unreliable for inclusion in our estimation of stigma (Cordes et al. 2017). Stigmatizing responses for the three remaining questions were as follows:

1. “No, I would not buy fresh vegetables from a shopkeeper or vendor if I knew that this person had HIV.”
2. “No, a female teacher who has the AIDS virus, but is not sick, should not be allowed to continue teaching in the school.”
3. “Yes, I would want my family member’s AIDS virus infection to remain a secret.”

Women exhibit higher levels of internalized and enacted stigma than men in Sub-Saharan Africa (Geary et al. 2014; Mugoya and Ernst 2014) and are considered critical pathways to reducing community-level stigma (Kelly et al. 2017). Additionally, stigma is likely to be underestimated by surveys (Kalichman et al. 2019; Maughan-Brown 2010). We therefore constructed a measure of stigma as the proportion of female respondents across Kenya who answered at least two questions of the remaining three (Questions 1, 3, and 4) in a stigmatizing manner, resulting in the time-ordered pairs of data:

$$\begin{aligned} (2003, 0.3622), (2008, 0.2654), (2014, 0.2654). \end{aligned}$$

We interpret these values as the fraction of Kenyans that have a stigmatizing view of HIV/AIDS irrespective of infection status and consider this to be a measure of population-wide stigma. Raw data used to calculate these values are given in “Appendix 1.”

We acknowledge the difficulty in accurately measuring stigma through surveys and the limitations of the KDHS questions, including bias from respondents indicating they do not engage in stigmatizing behaviors and concerns over how some questions may be understood by respondents (Cordes et al. 2017; USAIDS 2005; Yoder and Nyblade 2004). However, the KDHS questionnaire is the only study gathering standardized, national-level information on attitudes toward PLWHA for many countries at regular intervals over time and therefore allows us to consider the effects of stigma on a national population of PLWHA. Our estimates of stigma were validated by comparing our estimated values with findings of smaller studies within Kenya between 2003 and 2014 using more comprehensive instruments for measuring stigma (National Empowerment Network of People Living With HIV and AIDS in Kenya (NEPHAK) et al. 2011; Neuman and Obermeyer 2013).

### Creating a Mechanistic Model for Stigma in Kenya

We let $\sigma (t)$ represent population-level stigma as defined above and make the following model assumptions:

- There is a lower bound $\sigma _i$ for $\sigma (t)$ which corresponds to population levels of internalized stigma at equilibrium;
- The rate at which stigma changes in the population is directly proportional to the difference between current levels of stigma and the lower bound.

Combining these assumptions gives rise to the model equation:

1 $$\begin{aligned} \displaystyle \frac{\mathrm{d} \sigma }{\mathrm{d}t} = \nu (\sigma _i-\sigma ) \end{aligned}$$

where $\nu$ and $\sigma _i$ are positive constants and $\sigma (0)$ is specified. While simple in structure, this model still provides a caricature of the three components of stigma—internalized, enacted, and perceived—and allows us to determine the impact of interventions on each of these components:

- At equilibrium, $\sigma = \sigma _i$. Therefore, we interpret $\sigma _i$ as the population-level *internalized stigma;*
- The rate $\nu$ at which stigma changes over time represents how *enacted and perceived stigma* change due to external drivers such as advertising campaigns for HIV treatment.

Taking our starting point to correspond to the year 2000, we use the Curve Fitting Toolbox in MATLAB to obtain parameter estimates $\nu =0.24$, $\sigma _i=0.23$ and $\sigma (0)=0.5$. Using these values, we find the solution to (1),

2 $$\begin{aligned} \sigma (t) = 0.27 e^{-0.24( t -2000) }+0.23. \end{aligned}$$

**Fig. 1:**
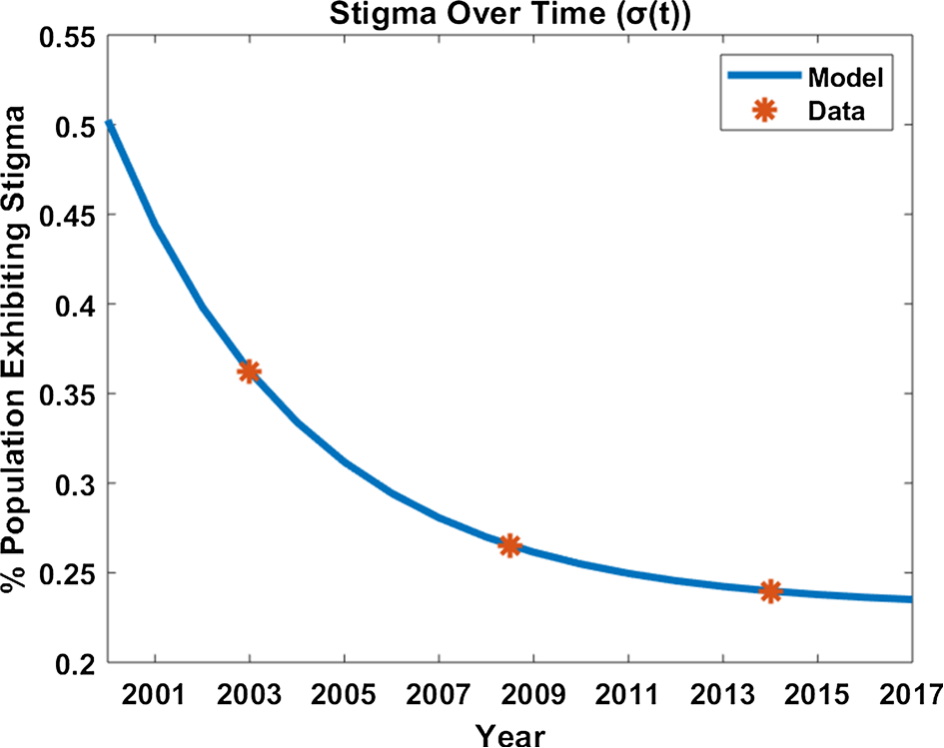
Fitted curve for $\sigma (t)$ (blue line) shown with data points (orange stars) from the three Kenya Demographic and Health Surveys. The starting point was chosen to correspond to the year 2000

See Fig. 1 for the fit of this function to the three data points from the KDHS. We consider this function of $\sigma (t)$ to be the primary analytical scenario representative of the observed levels of stigma in Kenya during 2003–2014. In the Results section, we use this fit as our baseline from which to consider alternative parameter values corresponding to different levels of internalized or enacted/perceived stigma.

## Modeling Infection Dynamics

Since 2000, the adult population in Kenya (16–64-year olds) has been growing just over $3\%$ per annum and so we cannot make the commonly used assumption for infectious disease modeling that there is a constant population. Rather, we assume that the adult population is growing exponentially (a good fit with the data as shown in the following section) and let *N*(*t*) denote the adult population in Kenya at time *t* (*t* measured in years). We use a compartmental structure for the population, assuming that there are two infected classes:

- $I_1(t)$ denotes individuals who are infected with HIV that are seeking treatment (individuals experiencing little or no impact from population-level stigma); and
- $I_2(t)$ denotes individuals with HIV that are not seeking treatment because they experience and are impacted by the population level stigma.

With this structure, we note that the number of individuals in the population that are not infected with HIV, the susceptibles *S*(*t*) can be calculated using the simple relation:

$$\begin{aligned} S(t) = N(t) -I_1(t) - I_2(t). \end{aligned}$$

**Fig. 2:**
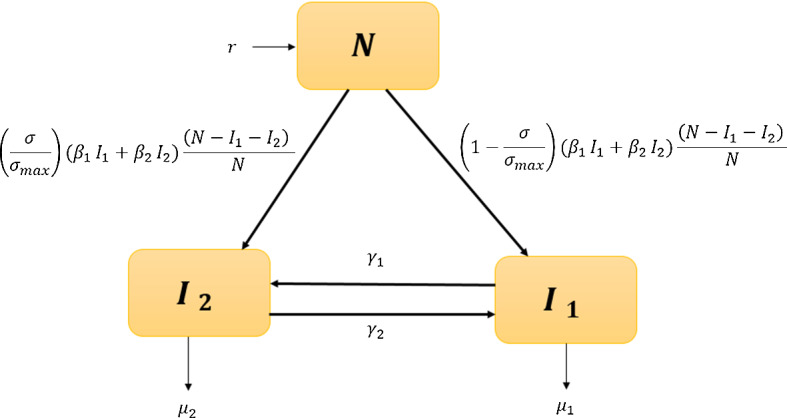
Schematics of the compartmental model

Following standard practice, we assume a frequency-dependent infection rate with individuals joining class $I_1$ or $I_2$ depending on the level of population stigma $\sigma$. Individuals may move between the two infected classes at rates dependent on $\sigma$ and may die from natural and/or disease related causes. Using these simple assumptions, together with (1), we obtain the model system of ODEs, shown also in the schematic presented in Fig. 2:

3a $$\begin{aligned} \frac{\mathrm{d}N}{\mathrm{d}t}&= rN \end{aligned}$$

3b $$\begin{aligned} \frac{\mathrm{d}I_1}{\mathrm{d}t}&=\left( 1-\frac{\sigma }{\sigma _{\max }}\right) (\beta _1I_1+\beta _2I_2)\frac{(N-I_1-I_2)}{N}-\gamma _{1}(\sigma ) I_{1}+\gamma _{2}(\sigma ) I_{2}-\mu _{1}I_1, \end{aligned}$$

3c $$\begin{aligned} \frac{\mathrm{d}I_2}{\mathrm{d}t}&=\frac{\sigma }{\sigma _{\max }}(\beta _1I_1+\beta _2I_2)\frac{(N-I_1-I_2)}{N}+\gamma _{1}(\sigma ) I_{1}-\gamma _{2}(\sigma ) I_{2}-\mu _{2}I_2, \end{aligned}$$

3d $$\begin{aligned} \frac{\mathrm{d} \sigma }{\mathrm{d}t}&= \nu (\sigma _i-\sigma ). \end{aligned}$$

with associated positive initial conditions for each variable. The parameter *r* represents the intrinsic growth rate of the population. The parameters $\beta _i$, $i=1, 2$ denote the transmission rates from individuals in compartment $I_i$, and $\mu _i$ is the corresponding death rate from those compartments (due to natural and disease-related causes). The parameter $\sigma _{\max }$ denotes the maximum impact of stigma on newly infected individuals, i.e., if $\sigma = \sigma _{\max }$ then all newly infected individuals will move into the $I_2$ class and will not seek treatment. Our model system is positively invariant and so our solution set will remain positive throughout, given the positivity of the initial conditions.

Movement between the two infected classes is represented by the rate functions $\gamma _i$ which satisfy the following properties:

- $\gamma _1$ is a convex increasing function of $\sigma$ such that as stigma increases in the population, the rate at which individuals move from the treated $I_1$ class to the untreated $I_2$ class increases;
- $\gamma _2$ is a convex, decreasing function of $\sigma$ such that as stigma increases in the population, the rate at which individuals move from untreated $I_2$ to treated $I_1$ decreases.

These choices were made in the absence of empirical data as parsimonious, based on the principle that the more prevalent stigma is within a population then the more likely individuals are to try and avoid seeking treatment for HIV and that this behavior would become more pronounced the higher the level of stigma (hence the assumption of convexity).

Our data-driven choice of exponential growth for the total population means that the model system (3) does not admit any non-trivial steady-state solutions. Moreover, the infection classes are not easily linked to infection data from Kenya and they do not correspond to standard measures of infection, such as infection prevalence. With this in mind, we chose to transform the model system to consider infection prevalence *P*(*t*) and the fraction of infected individuals seeking treatment *V*(*t*) using the relations:

$$\begin{aligned}\begin{array}{ll} P= \frac{I_1+I_2}{N}, ~~ V = \frac{I_1}{I_1+I_2}. \end{array} \end{aligned}$$

This transformation gives rise to the transformed (*P*, *V*) model system:

4a $$\begin{aligned} \frac{\mathrm{d}P}{\mathrm{d}t}&= P[(1-P)(\beta _1V+\beta _2(1-V))-\mu _1V-\mu _2(1-V)-r], \end{aligned}$$

4b $$\begin{aligned} \frac{\mathrm{d}V}{\mathrm{d}P}&= \left( 1-\frac{\sigma }{\sigma _{\max }}-V\right) (\beta _1V+\beta _2(1-V))(1-P)-\gamma _1V+\gamma _2(1-V)\nonumber \\&\quad +\,(\mu _2-\mu _1)V(1-V) \end{aligned}$$

4c $$\begin{aligned} \frac{\mathrm{d} \sigma }{\mathrm{d}t}&= \nu (\sigma _i-\sigma ) \end{aligned}$$

which we use in our model analysis, and simulations. Note that positivity of the values of $I_1$ and $I_2$ guarantees that *V* is well defined. We simulate our system using the parameters estimated in the next section, taking our starting point to correspond to the year 2004.

### Parameter Estimation

Data indicating that individuals did not begin to take up treatment for HIV/AIDS in Kenya until 2004 motivated us to set $t=0$ corresponding to the year 2004. We used published data (The World Bank, World Development Indicators 2019; Global Burden of Disease Collaborative Network 2018; The World Bank 2019), given in “Appendix A.1,” to estimate initial conditions:

$$\begin{aligned} N(0) = 19{,}881{,}691, P(0)=\frac{1{,}556{,}539}{ 19{,}881{,}691} \approx 0.08, V(0)=2, \end{aligned}$$

and the average yearly growth rate for the adult population in Kenya over this period,

$$\begin{aligned} r \approx 0.032. \end{aligned}$$

Since there is evidence that antiretroviral therapy can reduce transmission of HIV by up to 96% (Cohen et al. 2013, 2016), we take

$$\begin{aligned} \beta _1=0.1\beta _2. \end{aligned}$$

Studies also agree that antiretroviral treatment reduces the likelihood of death due to the disease by more than 50% (Kasamba et al. 2012; Violari et al. 2008), and so we impose the constraint

$$\begin{aligned} \mu _1\le 0.5\mu _2. \end{aligned}$$

Movement between $I_1$ and $I_2$ depends on the level of stigma that exists in society and therefore also change over time. As a result, we assume that $\gamma _1(t)$ and $\gamma _2(t)$ are both functions of $\sigma (t)$. For the purpose of parameter estimation and simulations, we chose

$$\begin{aligned} \gamma _1(t)=b\sigma (t)^2 \quad \text{ and } \quad \gamma _2(t)=c(1-\sqrt{\sigma (t)}) \end{aligned}$$

both of which satisfy the qualitative characteristics described in Sect. 3. A second, distinct pair of functions was also used to validate model results; details can be found in “Appendix A.3.1.”

Including the parameters embedded within the $\gamma _i(t)$ functions ($i=1,2$), we have 6 unknown values to estimate: $\beta _2$, $\mu _1$, $\mu _2$, $\sigma _{\mathrm{max}}$, *b*, and *c*. To do this, we used data from Kenya (2004–2017) given in “Appendix A.1” (The World Bank, World Development Indicators 2019; Global Burden of Disease Collaborative Network 2018; The World Bank 2019).

Details of the fitting algorithm and its goodness of fit are provided in “Appendix A.2.” The resulting parameter estimates (together with those obtained directly from the literature) are given in Table 1.

From the estimated parameters in Table 1, we note that our estimated death rates satisfy

$$\begin{aligned} \mu _1 \approx 0.31 \mu _2, \end{aligned}$$

which agrees with findings that antiretroviral treatment reduces death in adults by around 34% (Kasamba et al. 2012).

The parameter *b* was estimated as $2.09\times 10^{-7}$ (Table 1), resulting in very little flow from the treated class ($I_1$) to the untreated class ($I_2$).

**Table 1:** Parameters used in our model. We obtained the estimate for $r\approx 0.032$ and the relationship $\beta _1\approx 0.1\beta _2$ from the literature (Gapminder 2016; United Nations, Department of Economic and Social Affairs, Population Division 2019; Cohen et al. 2013). In cases where a parameter was estimated from data, we have provided the bounds used in the optimization problem

Parameter	Bounds	Estimated Value	Units
*r*		0.032	$\hbox {Years}^{-1}$
$\beta _1$		0.0082	$\hbox {Years}^{-1}$
$\beta _2$	[0 0.2]	0.082	$\hbox {Years}^{-1}$
$\mu _1$	[0.021 0.2]	0.021	$\hbox {Years}^{-1}$
$\mu _2$	[0.021 0.2]	0.068	$\hbox {Years}^{-1}$
$\sigma _{\mathrm{max}}$	[0 0.5]	0.50	None
*b*	[0 100]	$2.09\times 10^{-7}$	$\hbox {Years}^{-1}$
*c*	[0 50]	0.133	$\hbox {Years}^{-1}$

**Fig. 3:**
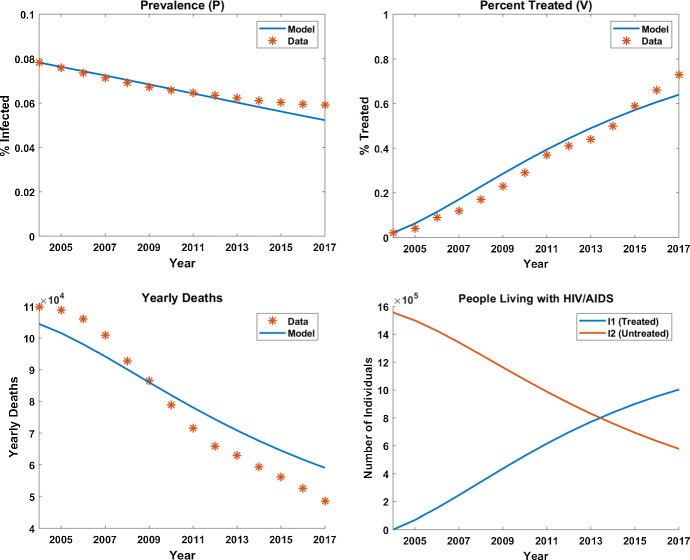
Model output compared to data after parameter estimation process

## Results

Figure 3 presents our model output using the estimated parameters together with the epidemiological data from Kenya. We explore how sensitive our model is to changes in “Appendix A.3,” which includes model simulation using alternative functional forms for $\gamma _1(t)$ and $\gamma _2(t)$. That work gives us confidence in the values of our disease-related parameters and confirms that the behaviors seen in the baseline case are qualitatively similar for alternative functional choices where we have no evidence or data on which to make our choices. Using our parameter estimates, the number of treated individuals ($I_1$) surpasses the number of non-treated individuals ($I_2$) in 2014, which agrees with the data. As time progresses, $\sigma (t)$ decreases allowing for an increasing percentage of new infections to begin treatment immediately. This accurately reflects what took place historically as the obtained data indicate that individuals in Kenya did not seek treatment for HIV/AIDS prior to 2000.

There are three distinct components of this results section. Firstly, we compare model outputs when parameter estimates associated with $\sigma (t)$ are varied from those described in Sect. 3.1. This allows us to understand better how stigma has impacted the HIV dynamics observed in Kenya over the period of interest. Next, we use model predictions to see how close Kenya might be to the UN “Getting to Zero” goal in 2030 and finally we undertake a standard steady-state analysis to explore the impact of long-term stigma on the fraction of PLWHA who seek treatment.

### Alternative Stigma Scenarios

**Fig. 4:**
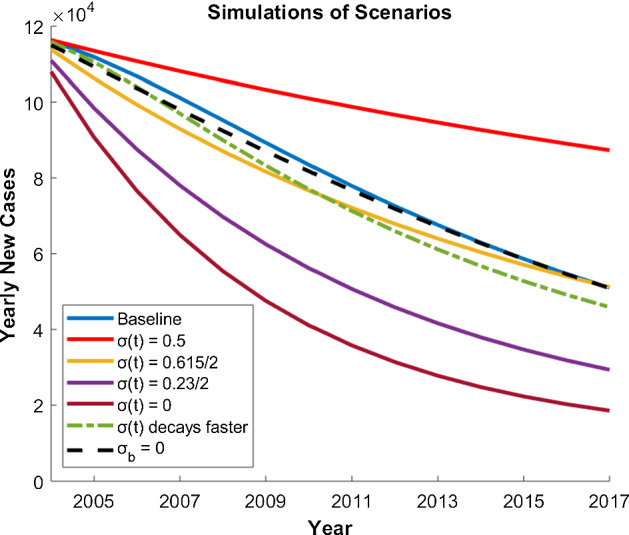
New cases 2004–2017 from the 7 scenarios described in Sect. 4. The baseline simulation uses our estimated parameters. We also simulated four scenarios where $\sigma (t)$ is constant over time, a case where $\sigma (t)$ decays twice as fast ($\nu =0.48$), and a scenario where we refit our $\sigma (t)$ function fixing internalized stigma as zero ($\sigma _i=0$ and $\sigma (t) =0.4e^{-0.04t}$)

We compare model predictions for the number of new cases each year, the total number of people being treated for HIV and deaths of people with HIV in the time interval 2004–2017, and we determine the time at which the models predict more infected people are seeking treatment than those who are not. We consider four scenarios that maintain $\sigma$ at a constant value; this can be interpreted as ignoring enacted stigma ($\nu =0$) but allowing population-wide internalized stigma to assume different levels, including the best-case scenario of no stigma. We consider one case in which $\nu$ is increased above the value estimated in Sect. 3.1 to explore the potential for a greater impact on reducing enacted stigma, and finally we compare these results to the case in which there is no internalized stigma assumed in the population $\sigma _i=0$. Results from these numerical explorations are presented in Fig. 4 and Table 2.

**Table 2:** Results of various scenarios all using the initial conditions from Sect. 3.1

Scenario	Switching month	Total deaths(millions)	Total treated(millions)
Baseline	113	1.13	1.04
$\sigma (t)=\sigma _{\mathrm{max}}=1/2$	298	1.31	0.60
$\sigma (t)=0.615/2$	104	1.09	1.02
$\sigma (t)=\sigma _{\mathrm{min}}=0.23/2$	60	0.91	1.25
$\sigma (t)=0$	41	0.79	1.34
$\sigma (t)$ decays faster ($\nu =0.48$)	99	1.08	1.09
$\sigma _i=0$ ($\sigma (t)=.4e^{-.04t}$)	113	1.12	1.04

Simulations are from 2004 through 2017. The switching month is defined as the month after January 2004 in which the percent of individuals that are being treated surpasses the percent of those not receiving treatment ($I_1(t)>I_2(t)$). Columns 3 and 4 display total values at the end of each simulation

#### Constant Stigma

Scenario 1 fixes the stigma level at its level in 2000 by setting

$$\begin{aligned} \sigma =\sigma _{\mathrm{max}}=1/2. \end{aligned}$$

This scenario considers what would occur if the National AIDS Control Council (NACC) was never formed in Kenya, resulting in a sustained stigma toward PLWHA.

In the second scenario, we let

$$\begin{aligned} \sigma =\sigma _{\mathrm{min}}=0.23/2. \end{aligned}$$

This corresponds to the stigma level starting and remaining at its lowest level, i.e., at its internalized stigma level.

The third scenario we considered corresponds to the midpoint of the previous two scenarios,

$$\begin{aligned} \sigma =0.615/2. \end{aligned}$$

In this case, $\frac{1}{\sigma _{\mathrm{max}}}*0.615/2=0.615$. According to our estimate of $\sigma (t)$, this midpoint occurred in 2005 and represents a scenario where the NACC formed, but did not effectively reduce stigma in Kenya resulting in 61.5% of new cases flowing into the untreated class for the duration of the simulation.

Lastly, we simulate the case where $\sigma =0$, representing a scenario where all stigma toward PLWHA is eliminated from society in Kenya so that all new cases immediately begin treatment. This hypothetical scenario may not be feasible, but it represents a “best case.”

There are clear differences in the results from the scenarios where stigma is constant over time. Note that in the case where $\sigma =\sigma _{\mathrm{max}}$, even though all new cases flowing into $I_2$ there is still movement from the untreated class ($I_2$) into the treated class ($I_1$). This simulation represents a “worst case” scenario where it takes nearly 300 months for the number of treated to surpass the number of untreated and over 1.31 million deaths occur after the 14-year simulation. In the simulations where $\sigma =0.615/2$, model output remains most similar to the baseline scenario of $\sigma (t)$ given in (2). In the cases where $\sigma =0.23$ and $\sigma =0$, we see significant decreases in the switching month and total deaths, as well as noticeable increases in the number of treated.

#### Stigma Decays Faster

Here, we simulate a scenario in which the rate at which stigma decays is twice that predicted using the stigma data from Kenya. Specifically, we set

$$\begin{aligned} \nu =2 \times 0.24=0.48. \end{aligned}$$

This represents a situation where public health education was more effective allowing for the perception of PLWHA to improve at a more rapid rate and can be thought of as a reduction in enacted stigma.

Although there was not a dramatic difference in most metrics from this scenario compared to the baseline simulation, allowing stigma to decay at a faster rate does result in slightly improved metrics across the board. We also note how the decrease in total deaths in this scenario is the same value as the increase in total treated. Thus, even though the number of yearly cases does not see a significant decline in this scenario (see Fig. 4), this highlights how a more rapid improvement in the perception of PLWHA (i.e., a reduction in enacted stigma) can save lives through more individuals seeking antiretroviral treatment.

#### No Internalized Stigma

Finally, we simulate a scenario where there is no internalized stigma, $\sigma _i=0$. In this case, we must first re-fit the function $\sigma (t)$ to the KDHS survey data using $\sigma _i=0$ because the solution trajectory that best fits the three data points we have estimated but for which $\sigma _i=0$, cannot be derived from our existing solution (2). The best fit solution gives

$$\begin{aligned} \sigma (t) = 0.4 e^{-0.04 t }. \end{aligned}$$

This case has similar output to the baseline scenario shown in Fig. 4, producing the same switching month and a slight reduction in total deaths. The reason for the similarity is that even though $\sigma (t) \rightarrow 0$ as $t \rightarrow \infty$, the baseline $\sigma (t)$ function (2) is not dramatically different during the simulated time frame. Having said that, the level of stigma at $t=0$ for this function is lower than that predicted with our baseline, data-driven estimate for $\sigma (t)$ and so more infected individuals move into the treated class early in the simulation resulting in a reduction in total cases and therefore also total deaths. Considering the clear importance of receiving treatment, allowing stigma to entirely dissipate from society would undoubtedly have a more significant impact when considering extended time frames.

### Meeting UN Goals

As described in the introduction, the UN initiative “Getting to Zero” aimed to reduce the number of new infections, the level of discrimination, and deaths from HIV/AIDS to zero by 2030. Our model predictions for these three measures are given in Table 3, where row 1 shows model output where an internalized level of stigma is assumed while row 2 assumes $\sigma _i=0$.

It should be noted that our model continues to assume exponential growth for the whole population until 2030 which certainly over-estimates the likely population in Kenya in 2030. That not with-standing, it is clear that there is likely to be a shortfall in achieving these goals. This is supported by our baseline model as output suggests that in 2030 about 23% of the population will stigmatize PLWHA, resulting in over 24,000 new cases and over 38,000 deaths that year. We obtain similar, though slightly lower, estimates in the case where the $\sigma _i=0$. Having said that, this is over $70\%$ reduction since 2003 and the reductions in the number of new cases and deaths by 2030 are considerable.

**Table 3:** Model output in 2030

Stigma level	Yearly new cases	Yearly deaths
0.231	24,427	38,448
0.120	21,500	36,364

Row 1 displays output from our baseline model while row 2 considers the case where $\sigma _i=0$ and $\sigma (t) = 0.4 e^{-0.04 t }$

### Understanding the Dynamics

**Fig. 5:**
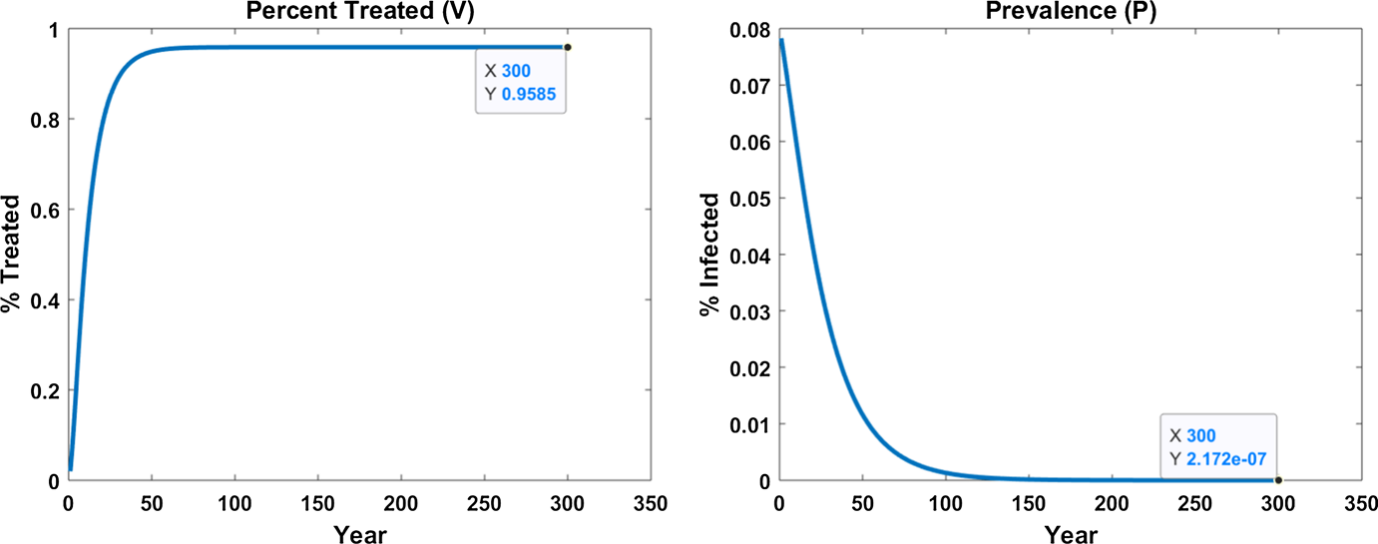
Simulation showing the long-term model predictions for (*P*, *V*) demonstrating the monotonic decline in prevalence to zero with around $96\%$ of PLWHA seeking treatment

Figure 5 shows the long term predictions of our model system using the parameter set fitted to Kenyan data. Our simulation predicts that as prevalence decays toward zero, the fraction of PLWHA seeking treatment stabilizes to a nonzero level. We use steady-state analysis as a proxy to explore this observation and find that it predicts that an infection-free equilibrium (zero prevalence) may arise under a range of conditions. At steady state, $\sigma ^* = \sigma _i$. When $P^*=0$, the corresponding equilibrium $V^*$ solves the quadratic equation:

$$\begin{aligned} A V^2 +BV +C = 0 \end{aligned}$$

where

$$\begin{aligned} A= & {} \beta _2-\beta _1+\mu _1-\mu _2 \\ B= & {} \beta _1 \left( 1-\frac{\sigma _i}{\sigma _{\max }}\right) -\beta _2 \left( 2-\frac{\sigma _i}{\sigma _{\max }}\right) +\mu _2-\mu _1-\gamma _1-\gamma _2\\ C= & {} \beta _2 \left( 1-\frac{\sigma _i}{\sigma _{\max }}\right) +\gamma _2. \end{aligned}$$

Of course, it is entirely unrealistic to assume that model parameters would remain unchanged over an extended period. What is interesting to glean from this analysis is a model estimate for the fraction of individuals infected with HIV that will seek out treatment (around 96 % in our particular parameter set for $V^*$ from that quadratic equation, and also matching with numerical predictions shown in Fig. 5). More details of our brief analysis are given in “Appendix A.4.”

## Discussion and Conclusions

Our motivation in undertaking the work presented here was to understand the impact of stigma on HIV prevalence in Kenya by developing and analyzing a mathematical model that could be adapted easily to explore HIV dynamics in other countries. We were careful to invoke Occam’s Razor such that the model parameters could be estimated using data readily available in the literature and where we did employ model assumptions, for example in determining how the population measure of stigma affected the movement of infected individuals between the treated and untreated classes, we validated the model outcomes by checking our results were independent of the particular functional form (provided it satisfied our baseline assumption).

Since the population in Kenya is growing at around $3\%$ per annum, one of our first challenges was to think about how to understand infection dynamics within a growing population. We addressed this by transforming the model system into one in which the state variables measured infection prevalence and the fraction of infected individuals seeking treatment. The next was to consider how stigma should impact the dynamics. With a paucity of data available to validate assumptions, we chose a parsimonious approach and decoupled the time evolution of stigma from the infection dynamics. Although the resulting model was simple in form, we were able to identify three critical components of stigma—internalized stigma and enacted plus perceived stigma—within the model. We used this to good effect in our analysis when we explored how changes to these two elements would have changed the amount of HIV infection in Kenya under a range of different scenarios. What emerged from that, as shown in Fig. 4, is that a reduction in internalized stigma (measured in the model by $\sigma _i$) would not have had a big impact on reducing incidence of infection in Kenya in the period 2004–2017; by contrast, reductions in enacted stigma would have reduced incidence by around $8\%$. Figure 4 also shows that if there was no stigma associated with HIV infections, then incidence of HIV infection would be lowest. This is hardly surprising. However, even in that scenario, the UN goal of Zero in 2030 would not be achieved according to our model predictions.

The model prediction that reducing enacted stigma might have more impact on reducing incidence of HIV than reducing internalized stigma provides a potential recommendation to those working in public health in Kenya. With limited resources available to tackle stigma, our model suggests that activities that target enacted stigma might be of greater benefit in the current situation than those targeting internalized stigma. This may be a welcome message—persuading communities to alter their view of HIV infection may provide a more tangible target for public health campaigns than initiatives that focus on individuals within those community.

It is clear that our model representation of stigma is simplistic. That was intentional for two reasons. Firstly, we did not find data-driven evidence in the literature that would link stigma dynamically to HIV infection dynamics. This meant that we could have chosen stigma to depend on infection prevalence, on HIV-related death, infection prevalence, and/or some combination of all of these. Our results may have been interesting but they may not have been relevant to Kenya. Secondly, we wanted to highlight the potential importance of incorporating stigma into our HIV model in order to support the argument that a focus on gathering data on sociological processes that impact infectious disease dynamics should start to take priority. While we move toward that goal, Kenya may not achieve the UN goal “Getting to Zero in 2030,” but it is certainly moving in the right direction.

## Figures and Tables

**Fig. 6:**
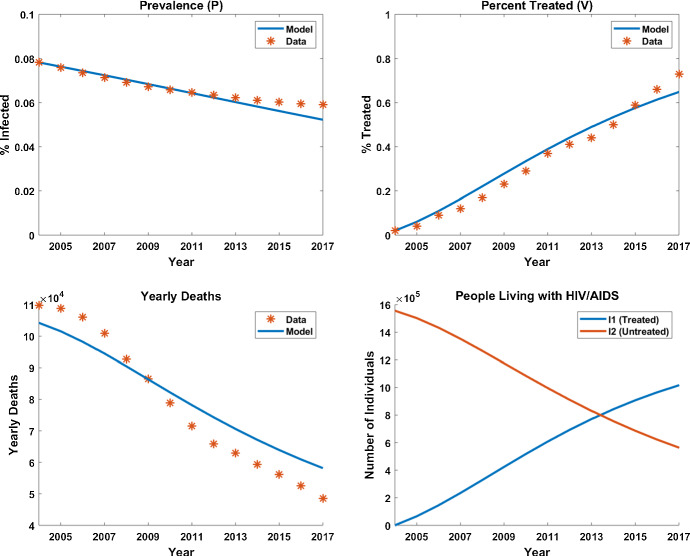
Model output compared to data after parameter estimation process using alternate forms for $\gamma _1(t)$ and $\gamma _2(t)$

## Appendix

### Data for Kenya

Data used to estimate key parameters $\beta _2$, $\mu _1$, $\mu _2$, $\sigma _{\mathrm{max}}$,*b*, and *c* include the fractions of Kenyans with a stigmatizing view of HIV/AIDS, i.e., the number of female respondents answering at least two questions in a stigmatizing manner divided by the total number of responses to all three questions (Table 4; CBS et al. 2004; Kenya National Bureau of Statistics (KNBS) and ICF Macro 2010, 2014), along with the total adult population, total number of HIV/AIDS cases, number of yearly HIV/AIDS-related deaths, and percent of PLWHA that are receiving antiretroviral therapy treatment for HIV/AIDS for Kenya from 2004 through 2017 (Table 5).

**Table 4:** Data for stigma used to estimate parameters in our model

Year	TFR for all three questions	SR for at least two questions
2003	8037	2911
2008	8349	2216
2014	14,633	3509

*TFR* total number of female responses, *SR* stigmatized responses

**Table 5:** Data for adult population (The World Bank, World Development Indicators 2019), total cases (Global Burden of Disease Collaborative Network 2018), yearly deaths (Global Burden of Disease Collaborative Network 2018), and percent treated (The World Bank 2019) used to estimate parameters in our model

Year	Adult population	Total cases	Yearly deaths	Percent treated
2004	19,881,691	1,556,539	109,769	2
2005	20,500,063	1,555,886	108,881	4
2006	21,134,459	1,556,425	106,011	9
2007	21,756,530	1,552,905	100,971	12
2008	22,384,120	1,549,630	92,753	17
2009	23,050,075	1,551,573	86,497	23
2010	23,771,983	1,564,451	78,894	29
2011	24,525,795	1,585,198	71,528	37
2012	25,345,229	1,607,145	65,842	41
2013	26,216,858	1,630,997	62,910	44
2014	27,117,617	1,657,895	59,355	50
2015	28,036,000	1,689,424	56,188	59
2016	28,988,590	1,727,026	52,482	66
2017	29,957,102	1,772,350	48,502	73

### Parameter Estimation Methodology

The total population together with the total cases gives the percentage of the population that is PLWHA. Using these data, we fit our model to the percent of the population that are a PLWHA ($PI^*$), the percent of the population that is a PLWHA and is receiving antiretroviral treatment ($PT^*$), and the number of yearly deaths caused by HIV/AIDS ($YD^*$). The asterisk represents data points while we use the equivalent notation without an asterisk to represent model output.

We estimate parameters by minimizing the objective function:

$$\begin{aligned} \text {minimize } J(\mathbf{x}) = \frac{||PI(\mathbf{x}) -PI^*||_2 }{||PI^*||_2}+\frac{||PT(\mathbf{x}) -PT^*||_2 }{||PT^*||_2}+\frac{||YD(\mathbf{x}) -YD^*||_2 }{||YD^*||_2}, \end{aligned}$$

where $\mathbf{x}^T = [ \beta _2, \mu _1, \mu _2, \sigma _{\max }, b, c]$ is the vector of unknown parameters from Table 1 (which also provides parameter bounds where these are known). Each vector comprises 14 components corresponding to the years, 2004–2017. We divide by the magnitude of the data to normalize each term.

The optimization problem was implemented in MATLAB using the fmincon function in the Optimization Toolbox. Parameter bounds and constraints that were imposed are detailed in the main text. Since fmincon is a local solver, we used MATLAB *multistart* to choose 200 starting points to fully explore the parameter space. All starting points converged to one of two local minimums, with the optimal set of parameters resulting in an objective function output of $J= 0.24$ and the parameter values shown in Table 1. The second local minimum produced an objective function value twice as large at $J=0.48$ and resulted in the same parameter values as the optimal set except that $b\approx 40$ rather than $b=2.09\times 10^{-7}$ and $c\approx 5$ rather than $c=0.133$. These values of *b* and *c* at the second minimum are unreasonably large, especially $b\approx 40$. More specifically, since $b\sigma (t)^2$ determines the percent of individuals moving from $I_1$ to $I_2$ per unit time, our model requires that $0\ge b*\sigma (t)^2\le 1$ where $0\ge \sigma (t)^2\le 1$. Thus, if $b\approx 40$ then $40\sigma (t)^2>1$ for $\sigma (t)>1/\sqrt{40}\approx 0.16$, which is problematic. Thus, the optimal set of parameters values are those given in Table 1.

### Sensitivity Analysis

We performed a global sensitivity analysis for our model using Latin Hypercube Sampling (LHS) to sample the parameter space and Partial Rank Correlation Coefficients (PRCC) to evaluate the sensitivity outcome variables of interest to changes in our 6 estimated parameter values. We created intervals for each parameters to sample from by extending 50% above and below the estimated values shown in Table 1. Uniform probability distributions were used for each parameter interval and we drew 100 samples from each interval. PRCC provides us with a way to evaluate the monotonicity of relationship of a parameter with each outcome variable of interest while holding all the remaining parameters constant, even when the relationship is not linear. We chose to evaluate the sensitivity of three outcome variables to changes in our parameters: Total Cases after the 14-year simulation, Yearly Cases in final year of the simulation, and Yearly Deaths in final year of the simulation (Blower and Dowlatabadi 1994; Marino et al. 2008). Results from this process are given in Table 6.

**Table 6:** Model sensitivity analysis results for three outcomes of interest: Total Cases after the 14-year simulation (*TC*), Yearly Cases in final year of the simulation (*YC*), and Yearly Deaths in final year of the simulation (*YD*)

	*TC*	*YC*	*YD*
$\beta _2$	$0.991^*$	$0.981^*$	$0.956^*$
$\mu _1$	$-\,0.043$	0.044	$0.842^*$
$\mu _2$	$-\,0.880^*$	$-\,0.893^*$	0.152
$\sigma _{\mathrm{max}}$	$0.833^*$	$0.809^*$	$0.835^*$
*b*	$-\,0.050$	$-\,0.053$	0.117
*c*	$-\,0.727^*$	$-\,0.821^*$	$-\,0.866^*$

Each entry represents the corresponding partial rank correlation coefficient (PRCC) and statistically significant values ($p<0.0001$) have an asterisk

Regardless of the variable of interest, our model is sensitive to changes in $\beta _2$, $\sigma _{\mathrm{max}}$, and *c*. While $\beta _2$ will determine the number of new infections per unit time, $\sigma _{\mathrm{max}}$ and *c* control the number of individuals that are in the treated ($I_1$) and untreated ($I_2$) classes. Death rate $\mu _1$ has a statistically significant impact on yearly deaths while $\mu _2$ is statistically significant with respect to the two outcome variables associated with number of cases.

#### Fitting Alternate Forms of $\gamma _1(t)$ and $\gamma _2(t)$

To determine how sensitive our parameter estimation results are to the functional forms of $\gamma _1(t)$ and $\gamma _2(t)$, we refit the model with the following functions that also satisfy the qualitative requirements stated in Sect. 3.1:

$$\begin{aligned} \gamma _1(t)= & {} b\sigma (t),\\ \gamma _2(t)= & {} \frac{c}{1+d\sigma (t)}. \end{aligned}$$

We followed exactly the process described in Sect. 3.1 and obtained the same parameter estimates as shown in Table 7 for the parameters not in the $\gamma _1$ and $\gamma _2$ functions. The corresponding simulation plots are depicted in Fig. 6. Additionally, we performed a sensitivity analysis on these parameters as described in “Appendix A.3.” The magnitude of the resulting PRCC values, their signs, and statistical significance closely aligned with those depicted in Table 6. The consistency of these results with those using the initial pair of functions suggests that we have reasonable estimates for our model parameters.

**Table 7:** Parameters used to test how sensitive our parameter estimation results are to the functional forms of $\gamma _1(t)$ and $\gamma _2(t)$

Parameter	Bounds	Estimated Value	Units
*r*		0.032	$\hbox {Years}^{-1}$
$\beta _1$		0.0082	$\hbox {Years}^{-1}$
$\beta _2$	[0 0.2]	0.082	$\hbox {Years}^{-1}$
$\mu _1$	[0.021 0.2]	0.021	$\hbox {Years}^{-1}$
$\mu _2$	[0.021 0.2]	0.068	$\hbox {Years}^{-1}$
$\sigma _{\mathrm{max}}$	[0 0.5]	0.50	None
*b*	[0 100]	$2.09\times 10^{-7}$	$\hbox {Years}^{-1}$
*c*	[0 50]	9.22	$\hbox {Years}^{-1}$
*d*	[0 500]	200	$\hbox {Years}^{-1}$

We obtained the estimate for $r\approx 0.032$ and the relationship $\beta _1\approx 0.1\beta _2$ from the literature (Gapminder 2016; United Nations, Department of Economic and Social Affairs, Population Division 2019; Cohen et al. 2013). In cases where a parameter was estimated from data, we have provided the bounds used in the optimization problem

### Steady State and Stability Calculations

At steady state, $\sigma ^* = \sigma _i$, and solutions of the transformed (*P*, *V*) system (4) satisfy the coupled algebraic equations:

5a $$\begin{aligned}&P[(1-P)(\beta _1V+\beta _2(1-V))-\mu _1V-\mu _2(1-V)-r] =0 \end{aligned}$$

5b $$\begin{aligned}&\left( 1-\frac{\sigma _i}{\sigma _{\max }}-V\right) (\beta _1V+\beta _2(1-V))(1-P)-\gamma _1V+\gamma _2(1-V)\nonumber \\&\quad +\,(\mu _2-\mu _1)V(1-V) =0. \end{aligned}$$

Solving (5) gives either $P^*=0$ or

6 $$\begin{aligned} P^*= & {} 1-\dfrac{\mu _1V^*+\mu _2(1-V^*)+r}{(\beta _1 V^*+\beta _2(1-V^*))}. \end{aligned}$$

We are interested in the equilibrium with $P^*=0$. This satisfies the first equation in (5); substituting into the second, we find that *V* must satisfy the quadratic equation:

7 $$\begin{aligned} A V^2 +BV +C = 0 \end{aligned}$$

where

8 $$\begin{aligned} A= & {} \beta _2-\beta _1+\mu _1-\mu _2 \nonumber \\ B= & {} \beta _1 \left( 1-\frac{\sigma _i}{\sigma _{\max }}\right) -\beta _2 \left( 2-\frac{\sigma _i}{\sigma _{\max }}\right) +\mu _2-\mu _1-\gamma _1-\gamma _2 \nonumber \\ C= & {} \beta _2 \left( 1-\frac{\sigma _i}{\sigma _{\max }}\right) +\gamma _2. \end{aligned}$$

Solving (7) gives the standard quadratic solutions

$$\begin{aligned} V_1^*= & {} \dfrac{-B+\sqrt{B^2-4AC}}{2A},\\ V_2^*= & {} \dfrac{-B-\sqrt{B^2-4AC}}{2A}. \end{aligned}$$

Since $C>0,$ the existence of equilibrium points is determined by the sign of *A*. Local stability of this infection-free equilibrium is determined by analysis of the Jacobian matrix

9 $$\begin{aligned} J = \left[ \begin{array}{cc} a_{11} &{} \quad 0 \\ a_{21} &{}\quad a_{22} \end{array} \right] \end{aligned}$$

where

$$\begin{aligned} a_{11}= & {} \beta _1 V^*+\beta _2 (1-V^*)-\mu _1 V^*-\mu _2(1-V^*)-r \\ a_{21}= & {} -\left( 1-\displaystyle \frac{\sigma _i}{\sigma _{\mathrm{max}}}-V^* \right) (\beta _1V^*+\beta _2(1-V^*)) \\ a_{22}= & {} -(\beta _1 V^*+\beta _2(1-V^*))+\left( 1-\displaystyle \frac{\sigma _i}{\sigma _{\mathrm{max}}}-V^* \right) (\beta _1-\beta _2)-\gamma _1-\gamma _2\\&+(\mu _2-\mu _1)(1-2V^*). \end{aligned}$$

Local stability is determined by the sign of the trace and determinant of *J*. Since this matrix is lower triangular, the stability conditions simplify to

$$\begin{aligned} a_{11}<0, \quad \text{ and } \quad a_{22} <0. \end{aligned}$$

For the parameter estimates, we have for Kenya, $A =\beta _2-\beta _1+\mu _1-\mu _2>0$. The scenario $A>0$ gives two possible positive roots for $V^*.$ We can show, by contradiction, that $V_1^*>1$ as follows. Assuming that $V_1^*\le 1$, then

$$\begin{aligned} -B+ \sqrt{B^2-4AC} <2A \end{aligned}$$

and hence

$$\begin{aligned} B^2-4AC < 4A^2+4AB+B^2. \end{aligned}$$

Substituting for *A*, *B* and *C* into this inequality gives

$$\begin{aligned} \dfrac{\sigma _i}{\sigma _{\max }}\beta _1+\gamma _1\le 0 \end{aligned}$$

which is a contradiction.

A direct manipulation on $$V_2^*$$ shows that $$0< V_2^*<1, $$ which gives the meaningful root for our model to get an equilibrium point $$(0,V_2^*).$$ Using the parameters values in Table 7, we obtain $$V_2^* = 0.9588$$ with $$a_{11}<0$$ and $$a_{22}<0$$ and so we conclude that this equilibrium is locally stable.

#### Remark 1

The case for $A<0$ leads to existence of one positive root of equation (5). An interior equilibrium point also exists when $P^*\ne 0$ and given by (6). Details for existence and stability of these equilibria have intentionally been omitted as we focus on the relevant equilibrium point predicted by fitting the model to data.
